# Release of Intracoronary Microparticles during Stent Implantation into Stable Atherosclerotic Lesions under Protection with an Aspiration Device

**DOI:** 10.1371/journal.pone.0124904

**Published:** 2015-04-27

**Authors:** Patrick Horn, Theodor Baars, Philipp Kahlert, Christian Heiss, Ralf Westenfeld, Malte Kelm, Raimund Erbel, Gerd Heusch, Petra Kleinbongard

**Affiliations:** 1 Division of Cardiology, Pulmonology, and Vascular Medicine, Medical Faculty, University Duesseldorf, Duesseldorf, Germany; 2 Institute for Pathophysiology, West German Heart and Vascular Centre Essen, University of Essen Medical School, Essen, Germany; 3 Clinic for Cardiology, West German Heart and Vascular Centre Essen, University of Essen Medical School, Essen, Germany; University of Munich, GERMANY

## Abstract

**Objective:**

Stent implantation into atherosclerotic coronary vessels impacts on downstream microvascular function and induces the release of particulate debris and soluble substances, which differs qualitatively and quantitatively between native right coronary arteries (RCAs) and saphenous vein grafts on right coronary arteries (SVG-RCAs). We have now quantified the release of microparticles (MPs) during stent implantation into stable atherosclerotic lesions and compared the release between RCAs and SVG-RCAs.

**Methods:**

In symptomatic, male patients with stable angina and a stenosis in their RCA or SVG-RCA, respectively (n = 14/14), plaque volume and composition were analyzed using intravascular ultrasound before stent implantation. Coronary aspirate was retrieved during stent implantation with a distal occlusion/aspiration device and divided into particulate debris and plasma. Particulate debris was weighed. Platelet-derived MPs (PMPs) were distinguished by flow cytometry as CD41^+^, endothelium-derived MPs (EMPs) as CD144^+^, CD62E^+^ and CD31^+^/CD41^-^, leukocyte-derived MPs as CD45^+^, and erythrocyte-derived MPs as CD235^+^.

**Results:**

In patients with comparable plaque volume and composition in RCAs and SVG-RCAs, intracoronary PMPs and EMPs were increased after stent implantation into their RCAs and SVG-RCAs (CD41^+^: 2729.6±645.6 vs. 4208.7±679.4 and 2355.9±503.9 vs. 3285.8±733.2 nr/µL; CD144^+^: 451.5±87.9 vs. 861.7±147.0 and 444.6±74.8 vs. 726.5±136.4 nr/µL; CD62E^+^: 1404.1±247.7 vs. 1844.3±378.6 and 1084.6±211.0 vs. 1783.8±384.3 nr/µL, P<0.05), but not different between RCAs and SVG-RCAs.

**Conclusion:**

Stenting in stable atherosclerotic lesions is associated with a substantial release not only of PMPs, but also of EMPs in RCAs and SVG-RCAs. Their release does not differ between RCAs and SVG-RCAs.

**Trial Registration:**

ClinicalTrials.gov NCT01430884

## Introduction

Stent implantation into an atherosclerotic lesion induces a traumatic plaque rupture and the release of particulate debris as well as of soluble vasomotor, thrombogenic, and inflammatory substances [1–4]. Both, the released particulate debris and the soluble substances contribute to impaired microvascular coronary perfusion [5–7]. The underlying atherosclerosis in native coronary arteries differs from that in SVGs, which is more rapidly progressing [8,9]. In patients with similar plaque volume and composition, stent implantation releases less particulate debris in native right coronary arteries (RCAs) than in SVGs on right coronary arteries (SVG-RCAs) [3].

Microparticles (MPs) are anucleoid phospholipid vesicles with a diameter between 0.1–1 μm. MPs are to be distinguished from smaller exosomes (0.04–0.1 μm), which originate from the endoplasmic membranes, and from larger phospholipid vesicles, the apoptotic bodies (>1.5 μm), which contain nuclear material [10]. MPs are shedded from plasma membranes of diverse source cells (endothelial cells and various blood cells, including platelets, leukocytes, and erythrocytes) [11,12] in response to various stimuli such as apoptosis [13], platelet activation [14], inflammatory cytokines e.g. tumor necrosis factor (TNF)α [15], and shear stress [16]. MPs contain a spectrum of bioactive molecules such as chemokines, cytokines, functional mRNAs and miRNAs, growth factors, and membrane receptors [11,17]. Platelet-derived MPs (PMPs) act as a source of vasoconstrictor thromboxane A_2_ and participate in thrombus formation and leukocyte adhesion [18]. Endothelium-derived MPs (EMPs) contribute also to impaired vasodilation [19].

MPs have been identified in human atherosclerotic plaques [20,21] and are increased in peripheral venous blood of patients with stable coronary artery disease (CAD) [12]. During a spontaneous plaque rupture in native coronary arteries i.e. in ST-elevation myocardial infarction (STEMI) the release of MPs in coronary blood was further increased [22–24] suggesting that plaque rupture and platelet activation might be a trigger/stimulus for MP formation. Restoration of epicardial blood flow led to reduction of intracororonary EMPs and PMPs [19,20] in this acute setting. MPs might serve not only as an index or marker of platelet activation or vascular injury, but also as a trigger of microvascular obstruction in patients with reperfused STEMI [25].

Whether or not a traumatic plaque rupture induced by a stent implantation into stable atherosclerotic lesions also induces a release of intracoronary EMPs and PMPs is not known. In the present study, we focused on patients with stable CAD undergoing elective stent implantation into stenotic RCAs or SVG-RCAs. We analyzed plaque volume and composition by intravascular ultrasound (IVUS) [6] before stent implantation. Stent implantation was done under protection with a distal occlusion/aspiration device, allowing us to capture the total released MPs into coronary blood during the traumatic plaque rupture. In the aspirated coronary blood we quantified the release of MPs and compared it between RCAs and SVG-RCAs.

## Methods

### Materials

Phycoerythrin (PE)-conjugated mouse anti-human cluster of differentiation (CD)144 antibody, fluoresceine isothiocyanate (FITC)-conjugated monoclonal mouse anti-human CD235 (glycoforin) antibody, PE-conjugated monoclonal mouse anti-human CD45 antibody were purchased from Beckman Coulter (Krefeld, Germany). FITC-conjugated monoclonal anti-tissue factor (TF) antibody was from Sekisui Diagnostics (Stamford, CT, USA). PE-conjugated mouse anti human CD62E, PE-conjugated mouse anti-human CD31 and PE-Cy5-conjugated mouse anti-human CD41 antibodies were from Beckton Dickinson Pharmingen (Heidelberg, Germany). Microbead standards were from Polyscience Inc. (Eppelheim, Germany), AccuCheck counting beads were purchased from Life Technologies (Darmstadt, Germany).

### Ethics Statement

The local institutional review board (Ethik-Kommission der Medizinischen Fakultät der Universität Duisburg-Essen; Germany, GZ.: 07–3387) approved this observational study. With patients’ written informed consent, we analyzed the coronary blood from symptomatic male patients with stable angina pectoris and with a stenosis in their RCAs or SVG-RCAs undergoing stent implantation under the use of a distal occlusion/aspiration device. We here respectively analyzed available aspirated coronary blood samples, which were leftovers from other prior studies [1,3,4,26,27]. Respective patients with a stenosis in their RCAs (n = 14) or SVG-RCAs (n = 14) were enrolled between March 2010 and May 2013. The study was conducted in accordance with the ethical guidelines of the Declaration of Helsinki 1975, and registered at ClinicalTrials.gov (ClinicalTrials.gov Identifier: NCT01430884).

### Quantitative coronary angiography

All patients were on oral aspirin (100 mg/day). Thirteen patients with RCA stenosis and 14 patients with SVG-RCA stenosis were also on oral clopidogrel (75 mg/day) prior to the intervention, for indications such as prior stent implantation or acute coronary syndromes within a year prior to the current study. After additional loading with clopidogrel (600 mg, oral) and heparin (10.000 I.U., i.v.), coronary angiography was performed using the femoral approach and 6 F or 8 F guiding catheters [1–4]. Stenosis severity was quantified using off-line caliper measurements (QCA-MEDIS, Leiden, NL) [28], and thrombolysis in myocardial infarction (TIMI) flow was measured before and after stent implantation [29].

### IVUS and virtual histology (VH)

IVUS was performed before and after stent implantation with a commercial catheter (Eagle-EyeTM 20 MHz Volcano Corporation, Rancho Cordova, CA, USA) and pullback device (0.05 mm/s, R-100, Volcano Corporation, Rancho Cordova, CA, USA). The site and length of the target lesion were retrospectively identified after stent implantation from landmarks in the vascular profile [1,30]. The plaque composition was categorized with VH using customized software (pcVHTM2.1, Volcano Corp.). Plaque components (fibrotic, fibro-fatty, necrotic core, dense calcium) were presented as a fraction of total plaque volume (%) [1,31].

### Interventional procedure

The implantation of balloon-expandable bare metal stents was performed with direct stenting and without prior dilatation/debulking using a stent-to-vessel diameter ratio of 1:1.15, because stenting with prior dilatation increases plaque mobilisation and debris embolism [32]. To prevent coronary microembolization, a distal balloon occlusion/aspiration device (GuardWire Temporary Occlusion & Aspiration System; Medtronic Inc., Minneapolis, MN USA) [33] was used. Before stent implantation, the balloon of the device was inflated at 2–4 atm with contrast agent. After stent implantation, the balloon catheter was removed, and the aspiration catheter loaded on the wire-balloon. During slow withdrawal of this catheter, the blood column was retrieved. Then, the distal wire-balloon was deflated [1–4].

### Coronary arterial blood and aspirate

Coronary arterial blood was taken through the aspiration catheter (10 mL into Heparin S-Monovette, SARSTEDT AG & Co, Nümbrecht, Germany) distal to the lesion before the stent implantation and served as control. Coronary aspirate (between 10 and 20 mL) was filtered ex vivo through a 40 μm mesh filter. The aspirate dilution by contrast agent was corrected for by reference to the hematocrit [1–4]. The released particulate debris was retained on the filter and weighed. The filtered coronary arterial and aspirate samples were immediately centrifuged (800 g, 10 min). Platelet-free plasma was obtained by two centrifugations of the plasma samples (10000 g, 5 min, each). Plasma and platelet-free plasma were quickly frozen in liquid nitrogen and stored at -80°C until further use.

### Tissue factor (TF), TNFα, and troponin I

We determined the plasma concentration of TF and compared it with the number of TF-bearing MPs; TNFα - as a potential trigger for MP release—was determined in coronary arterial and aspirate plasma using enzyme immunometric assay kits (American diagnostica inc, Stamford, USA for tissue factor; Cayman Chemical Company, Ann Arbor, USA for TNFα) [1].

Peripheral venous blood was taken before and between 6 and 48 h after stent implantation to determine serum troponin I [1,27]. Serum troponin I was measured using a specific 2-side immunoassay detected with the Dimension RxL Max Integrated Chemistry System (Siemens Healthcare Diagnostics, Eschborn, Germany).

### Characterization of MP subpopulations by flow cytometry

MP subpopulations were distinguished by flow cytometry according to the expression of established surface antigens, as described previously [34]. Briefly, platelet-free plasma samples were incubated for 30 min with fluorochrome-labeled antibodies or matching isotype controls and analyzed in a FACSVerse flow cytometer (Beckton Dickinson, Heidelberg, Germany). MP size was demarcated from particles with smaller size (exosomes) and larger size (apoptotic bodies). The threshold of 0.2 μm was set the technical inability of the flow cytometer to detect particles <0.2 μm [35], realizing that MPs with a size between 0.1–0.2 μm were not accounted for in this way. The microbead standard of 1.0 μm was used as upper limit. The total number of MPs was quantified and the homogeneity of the sample verified by addition of an internal counting standard with two fluorospheres (30 μL AccuCheck counting beads with 30300 beads) to each sample (300 μL). The detected numbers of both fluorosphere beads were compared with numbers provided by the manufacture. The counted MP number per μL was normalized to the fluorosphere beads and expressed as number(nr)/μL. The MP subpopulations were defined as follows: platelet-derived MPs (PMPs) were defined as CD41^+^, endothelium-derived MPs (EMPs) as CD144^+^, CD62E^+^ or CD31^+^/CD41^-^, leukocyte-derived MPs (LMPs) as CD45^+^, and erythrocyte-derived MPs (ERMPs) as CD235^+^. TF-bearing MPs were defined as MPs positive for TF antibody corrected by isotype background.

### Statistical analysis

Continuous data are presented as mean±standard error of the mean (SEM), categorical data as absolute numbers. Patient characteristics were compared using unpaired t test (continuous data) and 2-tailed Fisher’s exact test (categorical data). TF plasma concentration and the number of MPs in coronary arterial and aspirate plasma were additionally presented in a box plot as minimum and maximum (crosses), interquartile range from 25 to 75% (box), mean (square), and median (line). Data for serum troponin I before and after stent implantation, TF and TNFα plasma concentrations, and the number of MPs in coronary arterial and aspirate plasma were analyzed by a 2-way repeated measures ANOVA followed by Fisher’s LSD post-hoc tests. Differences were considered significant at the level of p<0.05. The statistics were done with SigmaStat, Systat software Inc., San Jose, USA.

## Results

### Patient characteristics, volume and composition of plaques

Patients with stenotic RCA and SVG-RCA, respectively, had comparable characteristics, apart from their target vessel, minimal lumen area, reference lumen area, and stent diameter (Tables 1 and 2). In both groups, TIMI flow was higher after stent implantation, but not different between groups. Serum troponin I was increased after stent implantation and also not different between groups (Table 3). However, the increase in troponin I after stent implantation under protection with an aspiration device was modest and exceeded the proposed cut off level of 0.15 μg/L, reflecting myonecrosis [36], in only 5 patients with stent implantation into RCA and 4 patients with stent implantation into SVG-RCA, respectively.

**Table 1:** Patient characteristics.

		RCA	SVG-RCA	P-value
**demographics**				
	number	14	14	1.0
	gender, female/male	0/14	0/14	1.0
	age [years]	58±3	64±3	0.2
	body height [cm]	176±2	176±2	0.9
	body weight [kg]	87±4	85±2	0.8
**risk factors/comorbities**				
	hypertension	14	14	1.0
	BMI [kg/m^2^]	28.1±1.4	27.4±0.7	0.7
	smoking	7	3	0.2
	diabetes mellitus	7	6	1.0
	hypercholesterolemia	13	14	1.0
	family history of CAD	6	5	1.0
**hemodynamics**				
	systolic blood pressure [mmHg]	129±7	130±7	0.9
	diastolic blood pressure [mmHg]	71±4	66±3	0.3
	heart rate [bpm]	63±3	67±3	0.3
**laboratory analysis**				
	erythrocytes [*10^12^/L]	4.5±0.2	4.6±0.1	0.7
	leukocytes [*10^9^/L]	7.4±0.5	7.1±0.5	0.9
	platelets [*10^9^/L]	226.2±19.8	212.2±12.6	0.5
	total cholesterol [mmol/L]	5.0±0.3	4.4±0.3	0.2
	HDL cholesterol [mmol/L]	1.1±0.1	1.0±0.1	0.5
	LDL cholesterol [mmol/L]	3.2±0.3	2.5±0.2	0.1
	triglycerides [mmol/L]	2.2±0.3	2.4±0.4	0.6
	serum creatinine [μmol/L]	145.2±30.4	136.4±34.1	0.8
	urea nitrogen [mmol/L]	7.7±0.9	7.5±1.2	0.9
	eGFR [mL/min/1.73 m^2^]	60.0±6.3	64.2±5.3	0.6
	HbA1c [%]	6.6±0.4	7.1±0.6	0.5
**medication**				
	ACE inhibitors	9	11	0.7
	AT1-receptor antagonists	2	1	1.0
	beta-blockers	12	13	1.0
	calcium antagonists	4	1	0.3
	statins	13	14	1.0
	diuretics	5	9	0.3
	antidiabetics	6	6	1.0
	insulin	1	0	1.0
	aspirin	14	14	1.0
	clopidogrel	13	14	1.0

Continuous data are presented as mean±SEM, categorical data as absolute numbers. Comparison between patients with RCA and with SVG-RCA by unpaired *t* test (continuous data) and 2-tailed Fisher’s exact test (categorical data). ACE = angiotensin-converting enzyme; AT1 = angiotensin II type 1; BMI = body mass index; bpm = beats per minute; CAD = coronary artery disease; eGFR = estimated glomerular filtration rate; HbA1c = hemoglobin A1c; HDL = high density lipoprotein; LDL = low density lipoprotein; RCA = native right coronary artery; SVG-RCA = saphenous vein graft on right coronary artery.

**Table 2:** Angiographic characteristics of the lesion.

		RCA	SVG-RCA	P-value
**number**		14	14	1.0
**lesion with thrombus**		0	0	1.0
**quantitative coronary angiography**				
	stenosis diameter [%]	63±3	60±4	0.6
**IVUS-analysis**				
	MLA [mm^2^]	2.9±0.1	4.0±0.5	0.03
	RLA [mm^2^]	6.9±0.6	10.9±1.7	0.02
	plaque burden [%]	68.1±2.4	64.9±2.9	0.4
**stent**				
	stent diameter [mm]	3.4±0.2	3.9±0.2	0.03
	stent length [mm]	21.4±1.2	18.8±0.8	0.1
**maximal balloon deployment pressure**				
	pressure [atm]	19.7±0.9	20.3±0.9	0.6

Continuous data are presented as mean±SEM, categorical data as absolute numbers. Comparison between patients with RCA and with SVG-RCA by unpaired *t* test (continuous data) and 2-tailed Fisher’s exact test (categorical data). IVUS = intravascular ultrasound; MLA = minimal lumen cross-sectional area in the culprit segment; RCA = native right coronary artery; RLA = reference lumen area; SVG-RCA = saphenous vein graft on right coronary artery.

**Table 3:** TIMI flow and serum troponin I before and after stent implantation.

	RCA		SVG-RCA	
	before	after	before	after
	stent implantation		stent implantation	
**TIMI flow n = 14 /14**	2.9 ± 0.1	3.0*	2.8 ± 0.1	3.0*
**troponin I [µg/L] n = 14 / 14**	0.0	0.6 ± 0.4*	0.0	0.7 ± 0.3*

Data are mean±SEM. Comparison by 2-way repeated measures ANOVA with Bonferroni’s correction;

^*^P<0.05 before *vs*. after stent implantation. RCA = native right coronary arteries; SVG-RCA = saphenous vein grafts on right coronary arteries; TIMI = thrombolysis in myocardial infarction.

### Volume and composition of plaques and particulate debris

**Fig 1:**
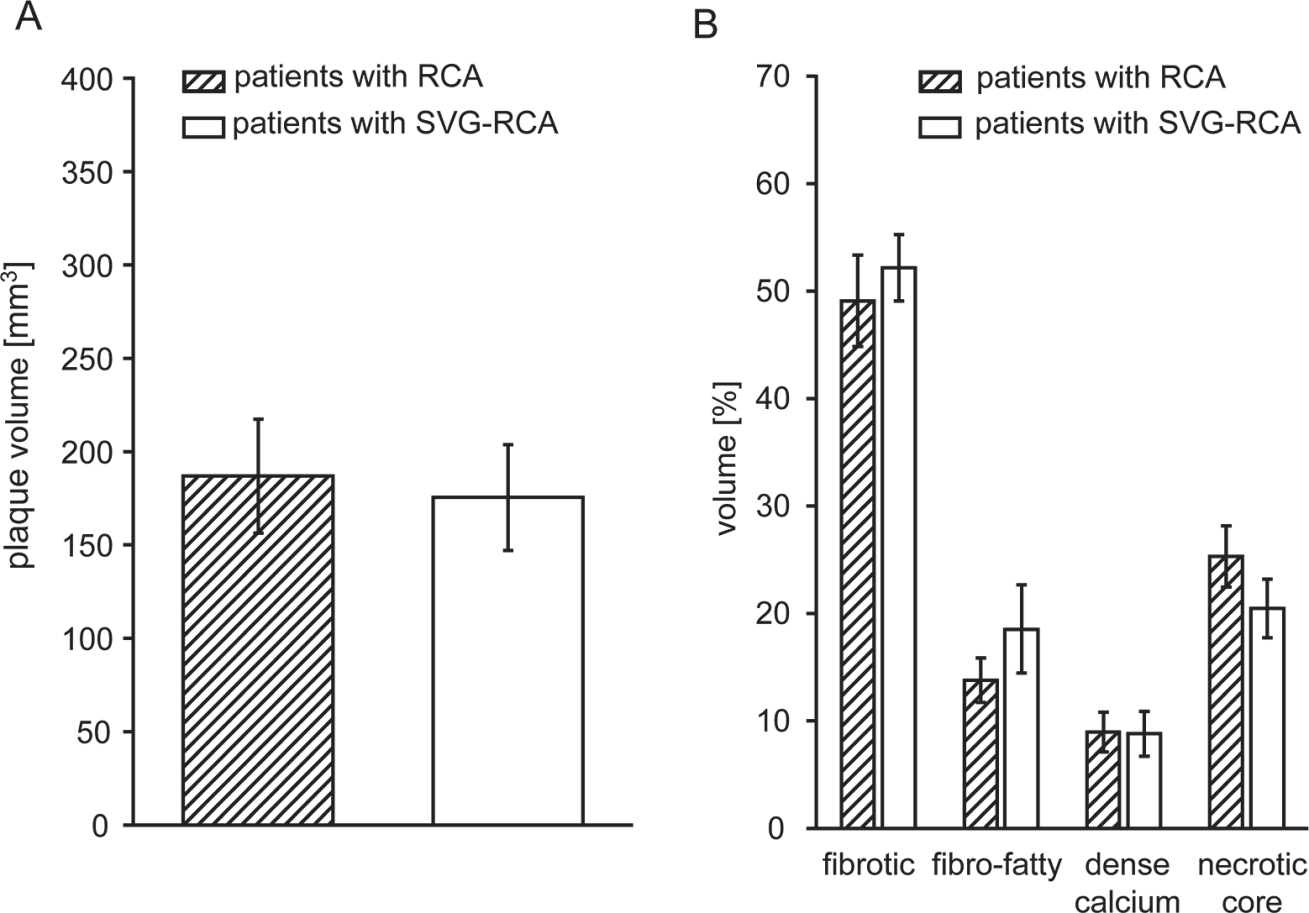
RCA and SVG-RCA plaque volume (A) and composition (B) by IVUS VH analysis. Data are mean±SEM, comparison by unpaired t-test. RCA = native right coronary arteries, SVG-RCA = saphenous vein grafts on right coronary arteries.

Plaque volume and composition were comparable between patients with a stenotic RCA and a stenotic SVG-RCA (Fig 1). The amount of released particulate debris from RCA was less than that from SVG-RCA, even when normalized to stent volume (0.30±0.09 vs. 0.87±0.17 mg/mm^3^, P<0.05).

### TF, TF-bearing MP, and TNFα in coronary aspirate

**Fig 2:**
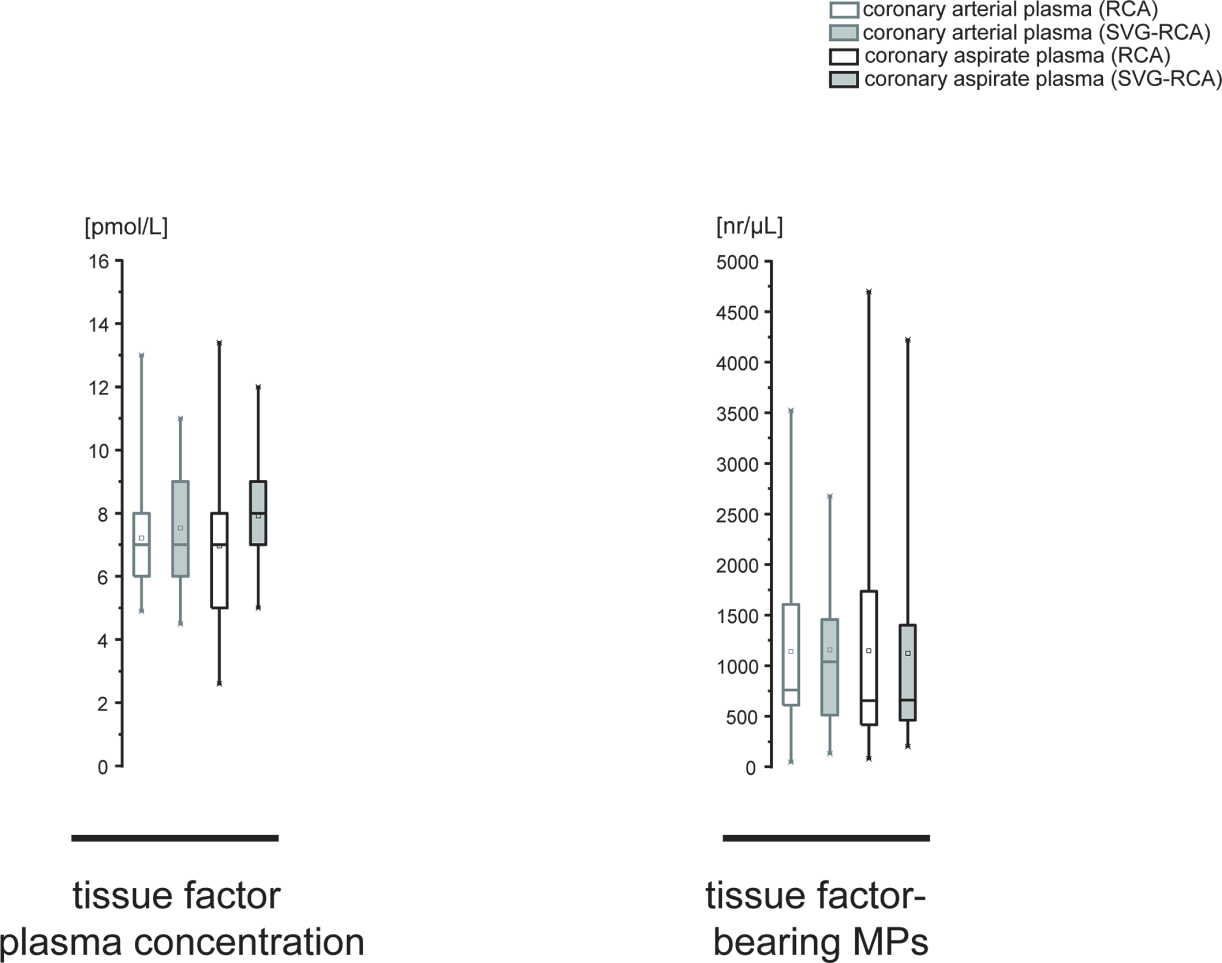
Plasma concentration of TF, and number of TF-bearing MPs in RCA and SVG-RCA before and after stent implantation. Data are presented as minimum and maximum (crosses), interquartile range from 25 to 75% (box), mean (square), and median (line) in a box plot. Comparison between arterial plasma and aspirate plasma as well as RCA and SVG-RCA was done by 2-way repeated measures ANOVA followed by Fishers LSD post-hoc test; MPs = microparticles, nr = number, RCA = native right coronary arteries, SVG-RCA = saphenous vein grafts on right coronary arteries, TF = tissue factor.

The plasma concentration of TF was not altered after stent implantation and not different between RCA and SVG-RCA (RCA: 7.2±0.6 vs. 7.0±0.8 pmol/L; SVG-RCA: 7.5±0.6 vs. 7.9±0.4 pmol/L, P<0.05). The number of TF-bearing MPs was also not altered after stent implantation within both groups and not different between groups (RCA: 1140.1±274.3 vs. 1148.1±339.6 nr/μL; SVG-RCA: 1155.6±207.3 vs. 1122.4±280.2 nr/μL, P<0.05) (Fig 2). The concentration of TNFα was increased after stent implantation in RCA and SVG-RCA (RCA: 0.4±0.05 vs. 1.7±0.3 pmol/L; SVG-RCA: 0.4±0.05 vs. 1.9±0.3 pmol/L, P<0.05), but not different between groups.

### MP in coronary aspirate

**Fig 3:**
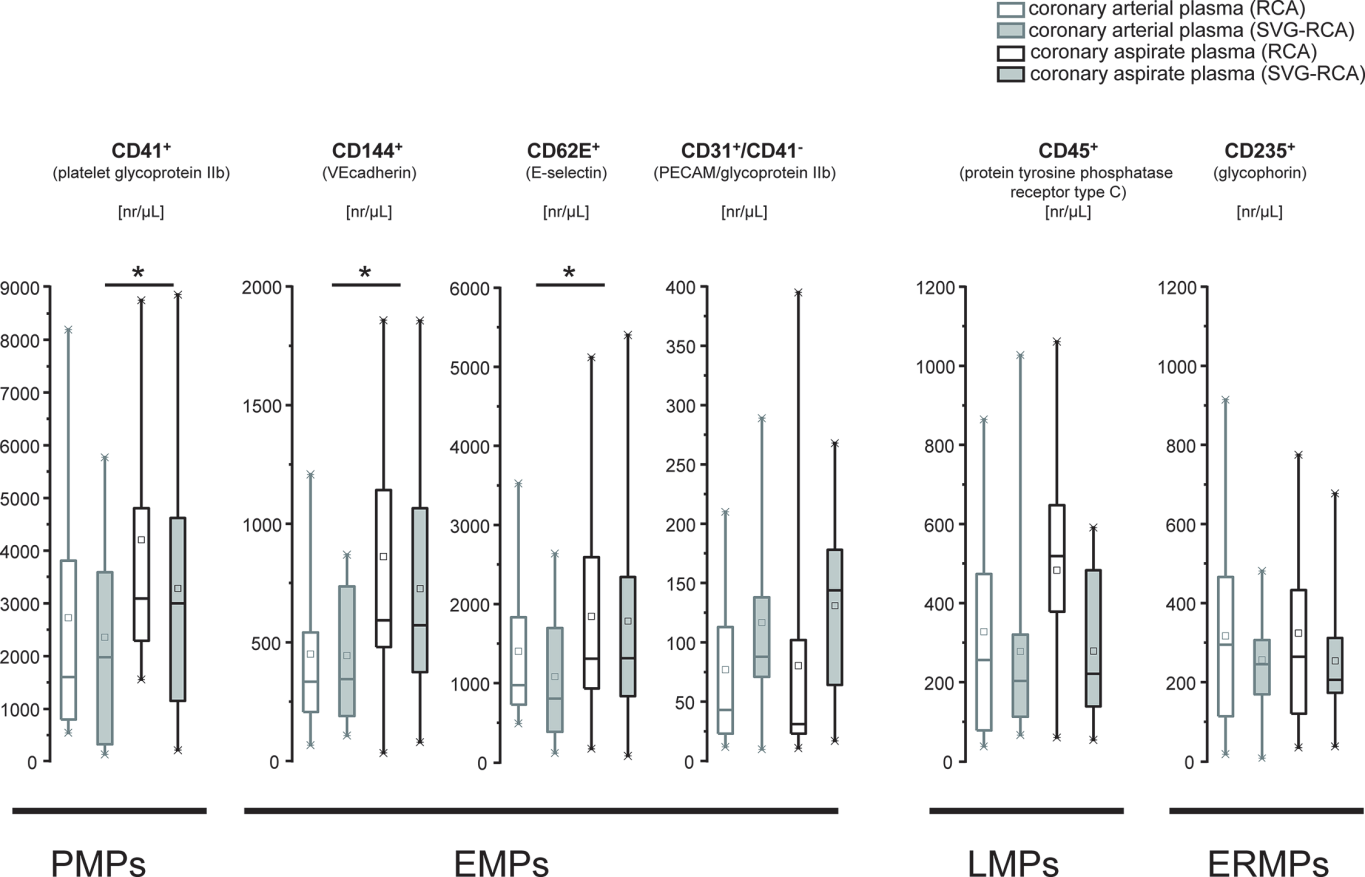
Numbers of MPs in RCA and SVG-RCA before and after stent implantation. Numbers of PMPs (CD41^+^), EMPs (CD144^+^, CD62E^+^ or CD31^+^/CD41^-^), LMPs (CD45^+^), and ERMPs (CD235^+^) are presented as minimum and maximum (crosses), interquartile range from 25 to 75% (box), mean (square), and median (line) in a box plot. Comparison between arterial plasma and aspirate plasma as well as RCA and SVG-RCA was done by 2-way repeated measures ANOVA followed by Fishers LSD post-hoc test; EMPs = endothelium-derived MPs, ERMPs = erythrocyte-derived MPs, LMPs = leukocyte-derived MPs, MPs = microparticles, nr = number, PMPs = platelet-derived MPs, RCA = native right coronary arteries, SVG-RCA = saphenous vein grafts on right coronary arteries.

The number of PMPs (CD41^+^) was increased after stent implantation in both groups, but not different between groups (RCA: 2729.6±645.6 vs. 4208.7±679.4 nr/μL; SVG-RCA: 2355.9±503.9 vs. 3285.8±733.2 nr/μL, P<0.05). The numbers of CD144^+^ and CD62^+^ were increased after stent implantation in RCA and SVG-RCA, but not different between groups (CD144^+^: 451.5±87.9 vs. 861.7±147.0 and 444.6±74.8 vs. 726.5±136.4 nr/μL; CD62E^+^: 1404.1±247.7 vs. 1844.3±378.6 and 1084.6±211.0 vs. 1783.8±384.3 nr/μL, P<0.05). The number of CD31^+^/CD41^-^ was not altered after stent implantation in both groups and not different between groups (RCA: 77.1±17.6 vs. 80.4±27.0 nr/μL; SVG-RCA: 116.7±20.3 vs. 130.9±19.3 nr/μL, P<0.05). The numbers of LMPs (CD45^+^) increased after stent implantation in RCA but not in SVG-RCA (327.7±74.2 vs. 482.8±77.5 and 278.1±63.8 vs. 279.1±49.3 nr/μL). However, the difference did not reach statistical significance. The numbers of ERMPs (CD235^+^) were not altered after stent implantation in both groups and not different between groups (317.3±64.7 vs. 324.0±56.6 and 256.6±43.3 vs. 254.2±43.2 nr/μL, *P<0*.*05*) (Fig 3).

## Discussion

We here identified and compared the release of MPs into the coronary blood during stent implantation into stable atherosclerotic lesions under protection with an occlusion/aspiration device. PMPs (CD41^+^), but also EMPs (CD144^+^ and CD62^+^) were released into RCAs and SVG-RCAs during stenting of stable atherosclerotic lesions. However, there was no difference between RCAs and SVG-RCAs. LMPs and ERMPs were not released during stent implantation.

In prior studies, the MPs extracted from human atherosclerotic plaques were mainly derived from activated leukocytes, erythrocytes and smooth muscle cells [20,21], whereas MPs in peripheral venous [37,38], systemic (arch of aorta) [24] and coronary blood [22,24,39] of patients with stable CAD or STEMI were mainly derived from platelets and endothelial cells [39]. Mechanical stimuli can trigger the sequestration of MPs from atherosclerotic plaques [20,21], vascular wall [38,40] and blood cells [39]. Stent implantation into the stable atherosclerotic lesion of RCA or SVG-RCA induced mechanical plaque rupture, and released different amounts of particulate debris. However, the numbers of MP released into RCA and SVG-RCA were comparable. Thus, MP release may not fully be explained as being a part of the particulate debris, but rather the generation of MPs from platelets and endothelial cells. In the present study, we confirmed prior reports by detecting a release of PMPs into the coronary blood of patients with stent implantation into stable atherosclerotic lesion [24,39]. For the first time, we identified also the release of EMPs into coronary blood after stenting a stable lesion, possibly as a result of the use of a distal balloon occlusion/aspiration device during stent implantation, which prevented the disappearance of MPs into the microcirculation. Numbers of LMPs increased after stent implantation in RCA, but not in SVG-RCA. The difference did not reach statistical significance at this point and future studies with a large number of biological samples will have to further address LMPs as a possible marker of the inflammatory response of stent implantation.

The precise molecular/biochemical trigger of MP generation from platelets and endothelial cells has not been validated in vivo, yet. Ex vivo, cell activation and apoptosis induce MP release by increasing intracellular calcium, loss in membrane lipid asymmetry, and cytoskeleton protein reorganization [12,40,41]. The inflammatory mediator TNFα stimulates the generation of EMPs in vitro from HUVEC after 24 h co-incubation [15]. Confirming our prior results [4], TNFα was released into the coronary aspirate, probably reflecting the inflammatory status of the vessel wall and promoting the generation and release of EMPs into the coronary aspirate *in vivo*. PMP generation is induced by platelet activation during percutaneous coronary intervention (PCI) [14,39]. Here, we detected PMP and EMP release during stent implantation of patients with stable CAD despite dual antiplatelet therapy. The dual antiplatelet therapy with aspirin and clopidogrel inhibits ADP-induced platetet activation and aggregation [42]. Clopidogrel is associated with decreased PMP and EMP numbers in patients with stable CAD [43,44]. Thus, in the present study the dual antiplatelet therapy possibly reduced, but did not prevented MP release.

In patients with STEMI undergoing PCI in their native coronary arteries [25], the release of MPs into coronary blood is accompanied by microvascular obstruction. PMPs promote thrombus generation [10] and might contribute to microvascular obstruction. In vivo- and in vitro-generated isolated EMPs decrease the production of nitric oxide when incubated with rat aortic rings ex vivo [45,46] and contribute to vasoconstriction. EMPs also promote inflammation in the vascular wall by enhancing pro-inflammatory cytokine release or leukocyte adhesion and migration [47]. MPs carry active TF and expose phospatidylserine on their surface, thus activating the coagulation cascade [48]. In animal studies, vascular injury induced by angioplasty of a stable atherosclerotic plaque results in increased TF expression of the vascular wall [49]. In patients with acute coronary syndromes, TF is released into the coronary blood [50]. In the present study, we measured the effect of elective stent implantation on TF in coronary aspirate of patients using two different methods: we determined TF-bearing MPs using FACS and measured the TF release into the aspirate using an immunometric assay kit. In both RCA and SVG-RCA, neither TF-bearing MPs nor plasma TF were increased after stent implantation. This is in line with previous studies demonstrating no increase of intracoronary plasma TF level immediately after stent implantation in stable CAD [1,3,4,26,51,52] and in acute myocardial infarction [22]. However, there is a delayed increase of TF in coronary sinus blood 24 h after stent implantation [53], possibly secondary to immunological or inflammatory processes. Independent of TF, MPs possess procoagulant activity [54].

Taken together, traumatic plaque rupture induced by stent implantation into stable atherosclerotic lesions triggers PMP and EMP release, which might contribute to microvascular obstruction after stent implantation. However, we were unable to analyze such microvascular obstruction or systemic effects, as—by definition—we removed MPs using the protection device; accordingly, post-interventional troponin release was only modest.

From a clinical view, the higher amount of particulate debris release in SVG-RCA intervention in our study may suggest a greater benefit of using protection filter device during intervention of a SVG as compared to intervention of a native coronary artery. In contrast, the comparable release of MPs between RCA and SVG-RCA suggest that strategies to reduce the deleterious effects of particulate debris and released MPs are likely to be equally important in both.

## Study Limitations

Here, we identified MPs released during stent implantation. However, this is a descriptive characterization of MP composition and their functional relevance remains unclear. Our study was limited to a small number of male patients undergoing elective stent implantation into their RCA or SVG-RCA, respectively, and requires prospective confirmation in larger cohorts. Furthermore, such a small sample size impedes the definitive explanation of the findings. IVUS VH has not been validated for use in SVG, and the lack of a clear interface between media and adventitia in SVG makes vessel volume measurements more problematic than in native coronary arteries [1]. The aspiration procedure of coronary blood before and after stent implantation might be an artificial trigger for MP formation and could have affected the measured MP number. In the present study the number of ERMPs did not change after stent implantation, possibly serving as a positive control. However, this control does not exclude artificial MP formations from other blood cells. Non-ionic contrast medium could also have induced platelet activation [55], and contributed to the PMPs count [39]. We did not confirm the MP release into coronary blood using an alternative method like electron microscopy. Furthermore, we did not use a parallel approach to characterize the MPs, e.g. PMPs with activation marker CD62P.
